# HIRA vs. DAXX: the two axes shaping the histone H3.3 landscape

**DOI:** 10.1038/s12276-023-01145-3

**Published:** 2024-02-01

**Authors:** Jinmi Choi, Taewan Kim, Eun-Jung Cho

**Affiliations:** Sungkyunkwan University School of Pharmacy, Seoburo 2066, Jangan-gu Suwon, Gyeonggi-do, 16419 Republic of Korea

**Keywords:** Molecular biology, Diseases

## Abstract

H3.3, the most common replacement variant for histone H3, has emerged as an important player in chromatin dynamics for controlling gene expression and genome integrity. While replicative variants H3.1 and H3.2 are primarily incorporated into nucleosomes during DNA synthesis, H3.3 is under the control of H3.3-specific histone chaperones for spatiotemporal incorporation throughout the cell cycle. Over the years, there has been progress in understanding the mechanisms by which H3.3 affects domain structure and function. Furthermore, H3.3 distribution and relative abundance profoundly impact cellular identity and plasticity during normal development and pathogenesis. Recurrent mutations in H3.3 and its chaperones have been identified in neoplastic transformation and developmental disorders, providing new insights into chromatin biology and disease. Here, we review recent findings emphasizing how two distinct histone chaperones, HIRA and DAXX, take part in the spatial and temporal distribution of H3.3 in different chromatin domains and ultimately achieve dynamic control of chromatin organization and function. Elucidating the H3.3 deposition pathways from the available histone pool will open new avenues for understanding the mechanisms by which H3.3 epigenetically regulates gene expression and its impact on cellular integrity and pathogenesis.

## Histone chaperones HIRA and DAXX: pioneers in chromatin organization control

Histones are key components of chromain, influencing DNA compaction, gene expression, and genome integrity. Unlike other histones, H3.3 is added to chromatin throughout the cell cycle by specific histone chaperones, namely HIRA and DAXX. This review explores how these two chaperones position H3.3 onto DNA, impacting the cellular management of genetic information and gene activity. Investigating the two axes of H3.3 deposition pathways underscores the delicate balance between HIRA and DAXX in saptial and temporal regulation of the H3.3 landscape from the available histone pool. Summarizing the effects of mutations on H3.3 and its chaperones in cancer and developmental disorders, the authors offer valuable insights into chromatin dynamics, development, and disease. This knowledge deepens our understanding of the complex interplay between H3.3 chaperones, providing insights into potential therapeutic strategies for diseases linked to H3.3 dysregulation.

This summary was initially drafted using artificial intelligence, then revised and fact-checked by the author.

## Introduction

The eukaryotic genome forms a chromatin structure that consists of regularly positioned nucleosomes along the genome. This structure enables cells to harbor ~3 billion bp long genomic DNA (in the case of human) packed within the nucleus and stably propagate while preventing the DNA from becoming twisted and tangled. Chromatin exists in a default condensed state that restricts the access of biological macromolecules and safeguards the genome. However, there are time- and space-dependent processes that open the chromatin structure and allow various biological functions, such as DNA replication, transcription, DNA damage repair, and recombination, to occur^1^. As a building unit for chromatin, the nucleosome core structure consists of 146 bp of DNA wrapped around a histone octamer assembled from the two copies of each of the core histones H2A, H2B, H3, and H4^2^. Histones regulate the chromatin structure through posttranslational modifications (PTMs), such as acetylation, methylation, phosphorylation, and ubiquitination^3^. Most nucleosomes in the cell are composed of these canonical histones. However, cells can express alternative forms of H2A, H2B, and H3, called noncanonical histone variants, which are incorporated into nucleosomes at specific genomic loci, indicating that they have distinct functions. The supply, exchange, deposition, and eviction of histones occur in DNA through the assembly and disassembly of nucleosomes, which is mediated by histone chaperones specific to canonical or noncanonical variants^4^. To ensure stable propagation of the genome and dynamic utilization of genetic information, histone chaperones play a pivotal role in chromatin dynamics and histone deposition on DNA. Here, we focus on two H3.3-specific histone chaperones that regulate the H3.3 landscape.

### H3.1/H3.2 vs. H3.3

**Fig. 1:**
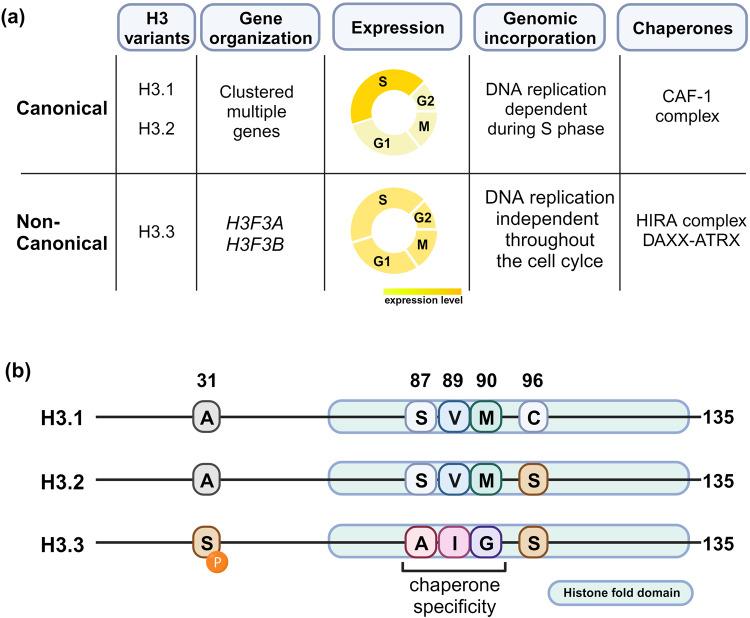
Three variants of histone H3. **a** The most common histone H3 variants include H3.1, H3.2, and H3.3. Canonical H3.1 and H3.2 are encoded by multiple clustered genes, while noncanonical histone H3.3 is encoded by *H3FA* and *H3FB*. Canonical histones are expressed and incorporated into the genome in a replication-dependent (RD) manner. On the other hand, H3.3 is expressed throughout the cell cycle and incorporated into the genome in a replication-independent (RI) manner. The integration of H3 variants into the genome is facilitated by distinct chaperone complexes: the CAF-1 complex for canonical histones and HIRA or DAXX/ATRX complexes for noncanonical H3.3. **b** The amino acid sequences are highly conserved among the three variants of histone H3. Notably, serine 31 in H3.3 establishes a distinctive phosphorylation site that is not present in H3.1 and H3.2. Chaperone specificity is determined by the AIG motif unique to H3.3.

Histone H3 has three representative variants that are evolutionally conserved: H3.1, H3.2, and H3.3 (Fig. 1a). Canonical forms (histone H3.1 and H3.2) are encoded from multiple copies of histone gene clusters, predominantly expressed during the S phase, and globally incorporated into DNA in a replication-dependent (RD) manner. RD-H3 deposition stably maintains nucleosome levels to ensure genome and epigenome integrity during cell division. In contrast, H3.3, a noncanonical variant, is encoded by two independent genes in humans, *H3F3A* and *H3F3B*, expressed throughout the cell cycle. H3.3 is incorporated into chromatin in a DNA replication-independent (RI) manner whenever histone turnover is necessary, such as during transcription and DNA damage repair^4,5^. Consequently, H3.1/H3.2 is used globally as a default substrate for chromatin synthesis and can be replaced by H3.3 at genomic locations where chromatin assembly and disassembly are actively occurring. In this manner, dividing cells are thought to maintain approximately 10–20% H3.3 nucleosomes, which increases to ~90% in postmitotic cells^6^. Although most H3.3 nucleosomes are assembled via the RI pathway, a recent study showed that preexisting H3.3 nucleosomes within the parental DNA strands can also be inherited by the daughter strands during S phase in an RD manner^7^. Yeast has one type of H3 that possesses characteristics from both H3.1 and H3.3. This H3 type is deposited through both the RD and RI pathways and plays dual functions.

H3.3 performs seemingly contradictory functions at different genetic loci. Initial studies discovered H3.3 within actively transcribed regions, promoting transcription through processes such as hyperacetylation of lysine (K) residues and methylation of H3K4 (H3K4me1/2/3), H3K36 (H3K36me3), and H3K79 (H3K79me3). Later studies revealed that H3.3 localized in heterochromatin suppresses transcription through processes such as K hypoacetylation and trimethylation of H3K9 (H3K9me3) and H4K20 (H4K20me3). Since gene expression programs are specific to the developmental lineage and differentiation status of cells, the H3.3 distribution profile can mirror distinct gene expression programs specific to different cell types (H3 barcode hypothesis)^8^. In this regard, the H3.3 mark is critical to maintaining cellular identity, acting as a roadblock that needs to be removed before differentiation and reprogramming can occur.

Although they have distinct functions, H3.3 differs from H3.1 by only five amino acids (S31, A87, I89, G90, S96) and from H3.2 by only four amino acids (S31, A87, I89, G90) (Fig. 1b). Three residues, A87, I89, and G90, known as the AIG motif, reside in the nucleosome core domain and determine the deposition route through direct contact with H3.3-specific histone chaperones^9–11^. In addition, H3.3-specific S31 phosphorylation (S31P) is important for maintaining chromatin and cell survival^12^. Interestingly, other residues known to undergo extensive PTMs for the regulation of chromatin functions are all conserved among variants. Two representative H3/H4 histone chaperones involved in the recognition and regulated deposition of H3.3 through the RI pathway are histone regulator A (HIRA) and death domain-associated protein (DAXX)^13,14^. In general, HIRA is involved in H3.3 deposition in euchromatin, such as actively transcribed gene bodies and regulatory elements, while DAXX governs H3.3 deposition in heterochromatin, such as pericentromeres, retrotransposons, and subtelomeric regions^14^.

There are several chaperones that load H3 onto DNA: CAF-1, NASP, and ASF1 for bulk nucleosome assembly and HJURP for the creation of specific chromatin domains. The CAF-1 complex consists of CHAF1A (p150), CHAF1B (p60), and RBBP4 (p48) in humans and participates in the assembly of nucleosomes using newly synthesized H3.1 and H3.2 during DNA replication and DNA damage repair^4,5^. NASP is involved in maintaining the soluble H3/H4 pool for histone homeostasis^15^. ASF1 (ASF1a, ASF1b) and NASP transport H3 from the H3/H4 reservoir to CAF-1 or HIRA without variant specificity. HJURP in humans is the histone chaperone of the centromere H3 (CenH3) variant CENP-A and is exclusively involved in the assembly of centromere nucleosomes^16^. The following sections summarize key findings on the dynamic regulation of H3.3 deposition and the control of chromatin functions via the HIRA and DAXX chaperones.

### HIRA vs. DAXX: two important players in the dynamic regulation of the H3.3 landscape

**Fig. 2:**
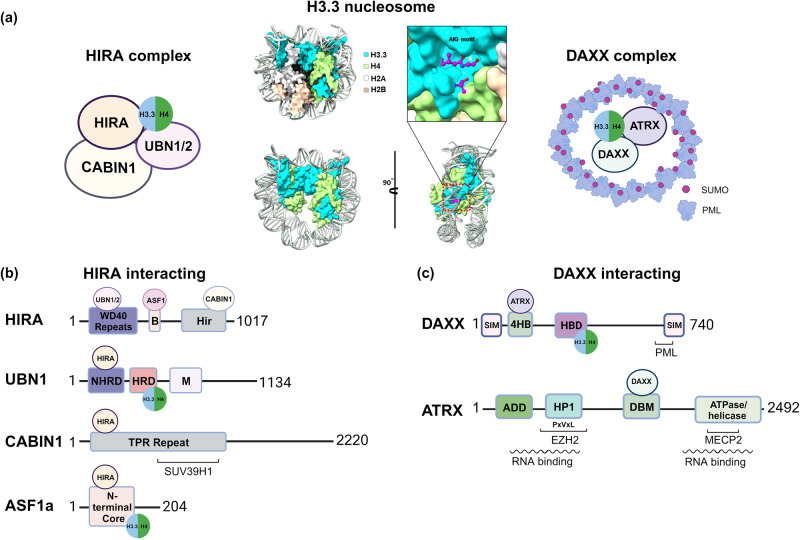
Characteristics of H3.3 and its chaperones, HIRA, and DAXX complexes. **a** Structure of the H3.3-containing nucleosome. Within the 5 amino acids that distinguish H3.3 from H3.1, the AIG motif (A87, I89, and G90) is recognized by UBN1/2 among the HIRA-interacting proteins or by DAXX. These variant-specific chaperones have distinct roles in depositing H3.3 in specific chromosomal regions: the HIRA complex contributes to H3.3 addition in euchromatin regions, while the DAXX/ATRX complex facilitates H3.3 deposition in telomeres, pericentromeric repeats, and other heterochromatic areas. **b** The functional domains of HIRA-interacting proteins for their chaperone activity. H3.3 interacts with the HRD and N-terminal core domains of UBN1 and ASF1, respectively. **c** The functional domains of ATRX and DAXX. H3.3 interacts with the HBD of DAXX. WD40 repeat Trp-Asp 40 repeat, Hir histone regulatory, NHRD nucleotide-binding and helical repeat domain, HRD helical repeat domain, M middle domain, TPR repeat tetratricopeptide repeat, SIM sumo-interaction motif, 4HB four-helix bundle, HBD histone-binding domain, ADD domain ATRX-DNMT3-DNMT3L domain, HP1 HP1-binding motif (PxVxL).

HIRA and DAXX are two principal chaperones that mediate the selective incorporation of H3.3 into nucleosomes in defined chromatin domains (Figs. 1a and 2a). Both chaperones assemble nucleosomes independently of DNA synthesis and act as a component of a multisubunit complex while interacting with various auxiliary proteins. They mediate the targeted incorporation of H3.3/H4 into nucleosomes at disparate locations with different outcomes. Furthermore, many studies have raised the possibility that these two chaperones may collaborate, complement, or compete with one another, as they utilize the same histone substrate from the limited pool of H3.3. Upstream biological signals and interactions with protein partners, noncoding RNAs, or genomic features might contribute to the elaborate regulation and balance of these two representative H3.3 deposition chaperone proteins.

#### HIRA complex

The HIRA complex is composed of HIRA, Calcineurin-binding protein 1 (CABIN1), and Ubinuclein 1 or 2 (UBN1/2) and cooperates with ASF1 to deposit H3.3^17–19^ (Fig. 2a, b). The HIRA complex may not necessarily be constructed through a 1:1 stoichiometric interaction. Recent biochemical and structural analysis showed that the HIRA subunit forms a stable homotrimer in vitro that interacts with two molecules of CABIN1 in a 3:2 stoichiometry^20^. Three functionally important domains of the HIRA protein are involved in H3/H4 binding and chromatin remodeling. Specifically, the N-terminus of the HIRA protein contains the WD40 domain, which is made up of seven WD40 repeats forming a beta-propeller structure. This WD40 domain has been shown to mediate histone binding by interacting with UBN1/UBN2, which recognize H3.3, and RBBP4 (p48), which binds H3/H4 within CAF-1 complexes as well^21^. The central B domain of HIRA is responsible for binding to the H3/H4 chaperone ASF1. The interaction between the B domain and ASF1 is crucial to recruiting ASF1 to chromatin and facilitating the incorporation of H3/H4 into newly formed nucleosomes. The Hir (histone regulatory) domain in the C-terminus is responsible for the interaction with CABIN1^21^. Domain features show that HIRA plays a central role in recruiting these proteins to chromatin and promoting chromatin remodeling through H3.3/H4 deposition. In addition, HIRA and UBN1/2 possess DNA-binding activities that might facilitate histone deposition on nucleosome-free DNA as a gap-filling mechanism^22–24^. Moreover, the chaperoning activity and subnuclear localization of HIRA can be modulated by various PTMs. For example, HIRA phosphorylation can cause its translocation to promyelocytic leukemia protein (PML) nuclear bodies (PML-NB) upon the initiation of human primary cell senescence^25^. HIRA phosphorylation also regulates H3.3 deposition during myogenesis^26^. The O-GlcNAcylation of HIRA is linked to the efficiency of H3.3 nucleosome assembly^27^. HIRA is also polyADP-ribosylated for the maintenance of telomeric chromatin under certain conditions^28^.

UBN1 and UBN2 are paralogs that play both similar and distinct roles in gene regulation and are alternatively present in the HIRA complex. Both UBN1 and UBN2 bind specifically to the H3.3/H4 dimer independently of the HIRA subunit through the evolutionarily conserved Hpc2-related domain (HRD)^10,11^. UBN1 and UBN2 specifically recognize A87 and G90 within the H3.3 AIG motif, endowing the HIRA complex with H3.3 specificity. The N-terminal domain (NHRD) of UBN1/2 is involved in the HIRA WD40 interaction, and the middle domain and its vicinity have UBN-UBN dimerization and DNA-binding activity, suggesting that UBN1 and UBN2 associate with DNA and mediate (H3.3/H4)_2_ tetramer formation prior to chromatin deposition^24^. It appears that UBN1 and UBN2-containing HIRA complexes can cooperate in depositing H3.3/H4 across the genome in transcriptionally active regions, such as cis-regulatory elements of neural developmental genes, during mouse embryonic stem cell (ESC) differentiation^11^.

CABIN1 was independently identified as a repressor of MEF2 and calcineurin-mediated signaling in immune cells^29^, but its function within the HIRA complex remains unclear. Considering that Cabin1 is a large protein of 2220 amino acid residues and is needed for complex stability, it could serve as a scaffolding platform for complex integrity^17^. Furthermore, given that CABIN1 is involved in the recruitment of mSin3/histone deacetylases (HDACs) and histone methyltransferases (HMTs), such as SUV39H1, in immune cells^30^, it could play a potential role in HIRA complex-mediated silencing and chromatin compaction. In fact, the H3.3 and HIRA complex is involved in the heterochromatinization of the genome in senescent human cells^17,31,32^.

#### ASF1 interacts with HIRA for H3.3 supply

ASF1 is an evolutionally conserved H3/H4 histone chaperone working at the interface of the RD and RI deposition pathways^5^. Yeast has a single type of Asf1, while in higher eukaryotes, ASF1 further diverges into ASF1a and ASF1b paralogs. ASF1b is responsible for supplying newly synthesized H3.1/H4 or H3.2/H4 dimers to the CAF-1 complex at the site of DNA replication, while ASF1a preferentially transfers new H3.3/H4 to HIRA and/or UBN1/2 by directly binding to the B domain of the HIRA protein^11,33^. The structural analysis of ASF1 suggested that it binds to the H3/H4 dimer without contacting the H3.3 AIG motif^34,35^. Although ASF1 itself does not provide H3 variant specificity, it is critical in linking H3/H4 to downstream chaperones, implying that the selection of H3 variants for transfer could be in part dependent on the abundance and availability of the H3 type from the histone pool. Furthermore, the ASF1-H3/H4 complex structure suggests that ASF1 is structurally incompatible with (H3/H4)_2_ tetramer binding, providing functional insights into its additional role in the disintegration of preexisting nucleosomes into H3/H4 dimers^34,36^.

Notably, each H3 binding protein (HIRA, UBN1/2) in the HIRA complex and ASF1 coordinately mediate the continuous flow of H3.3/H4 onto DNA. As HIRA and ASF1 do not have H3.3 specificity, UBN1/2 may be the determinant of H3.3 specificity, while ASF1 can contribute to widening the choice of H3 type. ASF1 may be responsible for splitting the (H3/H4)_2_ tetramer into H3/H4 dimers, allowing histone turnover through the simultaneous assembly and disassembly of nucleosomes. For example, the recycling of old H3.3 from preexisting nucleosomes is mediated by ASF1/HIRA during transcription^37^, implying that these chaperones could function in a highly coordinated manner to modulate the usage of old and new H3/H4 during polymerase II transcription^38^.

#### DAXX complex

DAXX is an H3.3-specific chaperone that cooperates with the alpha-thalassemia/mental retardation X-linked (ATRX) protein in RI nucleosome assembly. DAXX and ATRX are responsible for incorporating H3.3 into repetitive genomic regions such as pericentromeres, telomeres, and retrotransposons. Moreover, DAXX and ATRX modulate gene silencing and genome stability by chromatin compaction^39^. DAXX has several important domains that interact with many different protein partners, allowing it to participate in various cellular processes (Fig. 2c). First, DAXX has a highly conserved histone-binding domain (HBD) that provides H3.3 specificity^9,13,40^. Structural and biochemical studies have shown that the H3.3 AIG motif interacts with the DAXX HBD, whereby A87 is located in a shallow hydrophobic pocket, and G90 remains in the hydrophilic region of the HBD^9,13^. The DAXX HBD wraps around H3.3/H4 using an extended α-helical conformation, competing with histone–DNA and histone–ASF1 contacts, suggesting that the DAXX/H3.3/H4 complex prefers the H3.3/H4 dimer to the tetramer and is incompatible with cobinding with ASF1^40^. These hydrophobic, electrostatic, and hydrogen bond interactions contribute to stable complex formation; otherwise, DAXX could undergo destabilization upon loss of H3.3 contact^41^.

The N-terminal four helical bundle (4HB) domain is a structurally conserved motif that mediates protein–protein interactions, including ATRX. Specifically, the 4HB of DAXX interacts hydrophobically with the DAXX binding motif (DBM) of ATRX^41^. This interaction facilitates the formation of a stable DAXX–ATRX complex, which is then anchored to a H3K9me3-marked region for targeted H3.3 deposition into heterochromatin. The SUMO-interaction motif (SIM) at the N- and C-terminal ends interacts with SUMO-modified proteins. SUMOylation is the PTM in which the SUMO peptide is covalently attached to various target proteins, including PML. The SIM domain of DAXX binds specifically to SUMO-modified PML and contributes to its localization in PML-NBs^42^. Moreover, DAXX possesses an additional domain known as the PML-NB targeting region that aids in its localization. These characteristics ensure DAXX accumulation in PML-NBs and the formation of a stable DAXX/ATRX/H3.3 ternary complex.

ATRX is a main binding partner of DAXX, providing many heterochromatin-associated features. ATRX has two highly conserved domains: the ADD (ATRX-DNMT3-DNMT3L) and ATPase/helicase domains^43^. Many mutations associated with ATRX syndrome are predominantly located in these two domains, implying that the molecular function of ATRX largely depends on these domains. Importantly, ADD is responsible for the specific recognition of H3K9me3 in the presence of unmethylated H3K4 and the stable association with H3K9me3-marked chromatin domains^44^. The recruitment of ATRX to heterochromatin is further reinforced by interaction with HP1 through the PxVxL motif^45^. HP1 plays a crucial role in the lateral spreading of H3K9me3, chromatin compaction, and heterochromatin compartmentalization^46^. The ATPase/helicase domain is necessary for the translocation of ATRX along DNA using the energy of ATP hydrolysis. It alters DNA-histone contact within a nucleosome to facilitate the insertion of H3.3, in coupled with DAXX. Furthermore, ATRX directly binds MeCP2 through its C-terminal helicase domain, which is important for pericentromeric localization, heterochromatin organization^47^, and suppression of imprinting genes in the mouse brain^48,49^.

Importantly, ATRX can form facultative heterochromatin through H3K27me3, independent of DAXX. Polycomb repressive complex 2 (PRC2) comprises EZH2, EED, SUZ12, and RBAP46/48, where EZH2 is the HMT responsible for H3K27me3. ATRX recruits PRC2 through interaction with EZH2 for H3K27 methylation^50^. In addition, recent studies revealed that ATRX functions as an RNA-binding protein. Two RNA-binding domains are mapped on ATRX: one near the N-terminus and the other within the ATPase/helicase domain^50,51^. RNA binding contributes to the spatial location of ATRX and its binding proteins. For instance, during X chromosome inactivation (XCI), ATRX directly binds to Xist, a noncoding RNA (ncRNA), and mediates the spreading of EZH2-mediated H3K27me3 along the inactive X chromosome. Similarly, DAXX can function independently of ATRX. It suppresses endogenous retroviruses (ERVs) in mouse ESCs in a distinct complex containing SETDB1 (KMT1E), the corepressor protein KAP1 (TRIM28), and HDAC1^41^.

#### PML facilitates DAXX/ATRX-mediated H3.3 deposition

The PML body is a subnuclear structure comprised of a shell of PML proteins and many other proteins (>100) enriched inside^52^. PML bodies are associated with the buffering, storage, and PTM of various proteins and are involved in a wide range of cellular functions, including cell growth, apoptosis, antiviral resistance, senescence, and DNA damage responses^52^. In the nucleus, DAXX brings the newly synthesized H3.3/H4 dimer to PML-NBs, which is facilitated by ASF1A^53^. SUMOylation and the SUMO-SIM interaction between PML and DAXX lead to the accumulation of DAXX and ATRX in PML-NB^42,54^. H3.3/H4 is transferred to the DAXX/ATRX complex in PML-NB before loading onto defined chromatin domains^53,55,56^. Therefore, in chromatin biology, PML-NBs serve as an important insoluble compartment of H3.3/H4. Furthermore, PML is associated with heterochromatin, such as telomeres, and is involved in the maintenance of chromatin with H3K9me3 and H3K27me3^57^, emphasizing its role as a platform for the targeted supply of repressive H3.3 in these regions. Recent studies suggest that the fate of H3.3 is predetermined by the H3K9 methyl mark before nucleosome assembly. That is, DAXX promotes H3K9me3 in new H3.3/H4 prior to deposition via interaction with SUV39H1 and SETDB1 in a histone- and/or SUMOylated protein-dependent manner^58^, although whether this occurs in the context of PML-NBs remains to be revealed. Overall, the multitude of protein–protein interactions with the DAXX/ATRX complex ultimately promotes H3.3 loading and its role in gene silencing and chromatin compaction in repetitive genome regions with repressive histone marks such as K hypoacetylation, H3K9me3, H3K27me3, and H4K20me3.

### Distinct and overlapping functions of HIRA and DAXX in chromatin organization and gene regulation

#### Transcription-permissive chromatin and gene activation by HIRA complex

Several studies have demonstrated that HIRA and H3.3 nucleosomes are primarily associated with euchromatin and normal transcriptional regulation, including de novo transcription and long-term duration. In addition, the loss of HIRA and/or H3.3 has been linked to widespread transcriptional dysregulation and diminished recruitment of transcription factors. Meanwhile, chromatin accessibility either increases or decreases with the loss of HIRA/H3.3, depending on the genomic and cellular contexts^59–61^.

Many studies suggest that H3.3-containing nucleosomes are more susceptible to disruption than their conventional counterparts and become even more fragile when nucleosomes contain both H3.3 and H2A.Z^62–64^. H2A.Z is a variant of H2A that is incorporated into nucleosomes in an RI manner^63^. H3.3/H2A.Z double variant nucleosomes are enriched at highly transcribed gene bodies and regulatory regions such as promoters and enhancers^62–64^. This intrinsic susceptibility of the H3.3 nucleosome and/or the unfolding features of H3.3-containing chromatin structure may, in part, contribute to the dynamic control of chromatin accessibility to facilitate transcription factor binding and histone modifications that activate transcription^59,65^. However, given that both the HIRA complex and H3.3 cooperate with many other chromatin factors, the question remains to what extent such physical properties of the H3.3 nucleosome can contribute to chromatin states permissive to transcriptional events.

**Fig. 3:**
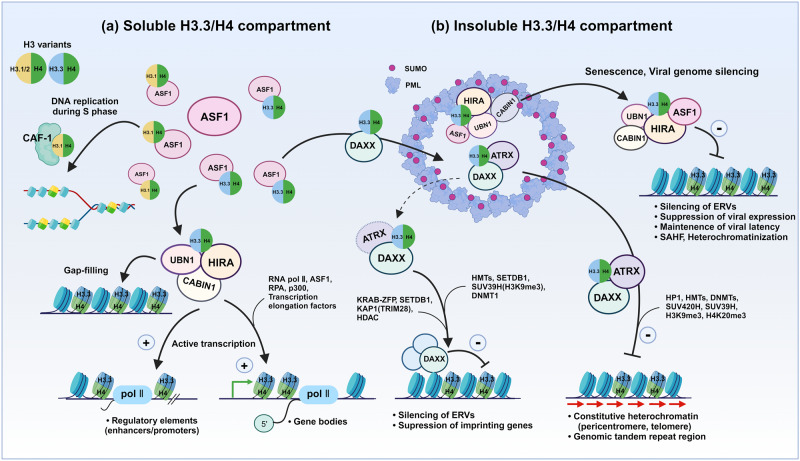
The various impacts of the H3.3 nucleosome on chromatin functions depend on the deposition route and its binding partners. ASF1 serves as the H3/H4 histone distribution hub in partnership with specific histone chaperones and partitions them into segregated histone compartments that are biochemically and functionally distinct. The operation of two distinct H3.3/H4 pools contributes to the determination of a subsequent loading route for H3.3 into specific chromatin regions to fulfill various functions. **a** ASF1 facilitates the transfer of newly synthesized H3/H4 from the soluble histone pool to either CAF-1 or HIRA complexes. CAF-1 incorporates H3.1/H4 or H3.2/H4 into nucleosomes during DNA replication in an RD manner. The HIRA complex can incorporate H3.3/H4 into nucleosomes in naked genomic regions (known as a gap-filling mechanism). HIRA specifically targets genomic regions such as transcription regulatory elements (promoters, enhancers) and gene bodies to mediate gene activation. **b** DAXX facilitates the accumulation of H3.3/H4 within PML-NBs, thereby maintaining the insoluble histone pool. Subsequently, DAXX/ATRX incorporates H3.3 into ERVs, imprinting genes, pericentromeres, and telomeres, contributing to gene silencing and genome stability. In addition, during senescence or viral infection, the HIRA complex is found within PML-NBs. HIRA-mediated incorporation of H3.3 via PML-NBs suppresses ERVs and viral expression through chromatin compaction. The localization of DAXX (and HIRA) in PML-NBs and the transition to genomic targets are largely affected by SUMOylation and SIM-SUMO interactions between proteins.

HIRA participates in multiple stages of transcription (Fig. 3a). During initiation, it interacts with a single-stranded DNA-binding protein, RPA, on promoters and enhancers to mediate H3.3 deposition and transcription^66^. The locally melted regulatory DNA and the transcription bubble are active sites of histone turnover and can be coupled to HIRA-mediated H3.3 deposition. Moreover, HIRA has been shown to couple with a SWI/SNF family chromatin remodeler, CHD1, during paternal pronuclear formation to ensure H3.3 nucleosome assembly and spacing^67,68^. Cooperation between HIRA and CHD1 also mediates H3.3 incorporation and global transcription in *Drosophila* adult brain cells^69^. Chd2, a closely related member of the SNF2 family, is also involved in the H3.3 marking of myogenic regulatory regions, where HIRA plays a role in muscle gene regulation and cell fate determination through H3.3 deposition^70,71^. The recruitment of HIRA through crosstalk with various transcription factors indeed enables the enrichment of H3.3 in promoters for gene activation^72^. Notably, H3.3 is needed for enhancer acetylation (H3K27ac) and activation of p300 during histone turnover, chromatin remodeler binding for transcription accuracy, rapid induction of inflammatory genes in mouse macrophages, mouse ESC differentiation, and *Xenopus* gastrulation^59–61,70,71,73^. HIRA is also involved in priming damaged loci by H3.3 deposition to facilitate transcriptional recovery after DNA damage repair^74^. These findings are consistent with high rates of H3.3 turnover in transcriptionally active sites^75^.

During elongation, HIRA binds to interferon-responsive genes to incorporate H3.3 into actively transcribed gene bodies^76^. HIRA occupancy was dependent on NSD2 (WHSC1), a H3K36 HMT that recruits BRD4 and the positive elongation factor P-TEFb to polymerase II, facilitating transcriptional elongation. Cooperation between H3.3K36me3 and S31P controls transcriptional elongation and RNA processing. For instance, ZMYND11 is a transcriptional corepressor that binds H3.3K36me3 within gene bodies and suppresses transcription elongation^77,78^. To promote transcription upon stimuli, H3.3 S31 is phosphorylated, which stimulates SETD2 activity, increases H3K36me3 levels, and displaces ZMYND11 for efficient gene expression in mouse macrophages^79^. During transcription elongation, the HIRA complex travels throughout the entire gene body together with polymerase II^22,76^. Indeed, HIRA KO decreases the H3K36me3 level, serine 2 phosphorylation of the polymerase II C-terminal domain (CTD), and the recruitment of elongation factors (ZMYND11, BRD4, CDK9) at the IgH locus in human B cells^61^. Altered CTD phosphorylation could affect transcription and mRNA processing^80,81^. Moreover, the polymerase elongation rate over chromatin structure can influence the cotranscriptional regulation of splicing^82^. Therefore, it is intriguing that the H3.3 level affects polymerase II elongation and cotranscriptional splicing and is associated with altered exon usage and intron retention after impairment^83^.

#### Heterochromatinization and gene silencing by DAXX/ATRX

DAXX/ATRX and H3.3 nucleosomes have been linked to gene silencing and constitutive heterochromatin formation in repetitive genomic regions (Fig. 3b). These repetitive DNA elements are heterochromatinized early during embryonic development to protect the genome from aberrant transcription and recombination. The ADD domain of ATRX and its interaction with HP1α facilitate DAXX-mediated H3.3 deposition and subsequent methylation of H3K9 by SETDB1 and SUV39H1/2 at these loci. The intermolecular interactions of HP1α-SUV39H-H3K9me3 promote the expansion of the heterochromatin domain. Although the precise role of the H3.3 nucleosome in these repressive chromatin contexts is largely unknown, it may affect heterochromatinization by permitting the limited local transcription of repeat elements. It is well-documented that many chromatin-associated proteins rely on ncRNAs for their proper localization^84^. Despite its heterochromatic nature, a small amount of RNA is transcribed from the pericentromeric region, which in turn facilitates heterochromatin retention of SUV39H1/2^85–87^ and HP1α^88,89^ to maintain the heterochromatin state. H2A.Z, which forms labile double variant nucleosomes with H3.3, was also shown to cooperate with HP1α to regulate heterochromatin domains^90^. ATRX, MeCP2, and satellite RNA transcripts cooperate for heterochromatin organization in neurons^47,91^. H3.3 can thus be associated with the local transcription of repeated regions. Along this line, accumulation of DAXX/ATRX in chromocenters and concomitant induction of pericentromeric transcripts are evident with pericentromeric/centromeric DNA clustering during myogenesis^92,93^ and neural differentiation^47,91^. Recent studies on biomolecular condensation have applied the concept of phase separation to the process of heterochromatinization of genomic repeats, where many proteins with internally disordered regions (IDRs), RNA binding, and RNA transcripts play a pivotal role through transient, weak, and multivalent intermolecular interactions^94^.

H3.3 is involved in the silencing of endogenous retroviral elements (ERVs) in mouse ESCs^95,96^^.^ ERVs such as MLV, IAP, and MusD are enriched with H3.3 and marked by H3K9me3 and H4K20me3, where their silencing is dependent on H3.3, SETDB1, and KAP1. SETDB1/KAP1 is independently known to cooperate with Kruppel-associated box zinc finger proteins (KRAB-ZFP) to silence transposable elements and imprinting genes based on the DNA-binding ability of KRAB-ZFP^97^. These reports showed that the recruitment of DAXX, H3.3, and KAP1 is codependent and required for H3K9me3 and subsequent repression of ERV expression and transposition, suggesting a functional interplay between H3.3 loading and H3K9me3 for heterochromatin establishment in ERVs^95,96^. Morc3 ATPase was also discovered by genome-wide screening, showing that it is an upstream regulator of H3.3 loading and ERV silencing through SUMO-SIM-mediated DAXX interaction^98^. However, it is unclear whether H3.3 nucleosomes and the chaperoning activity of DAXX are absolutely needed for the heterochromatinization of ERVs. Given ATRX-independent ERV suppression and the effect of H3.3 on DAXX stabilization^41^, the H3.3 profile might coincide with H3K9me3 without direct involvement of H3.3 in repressive nucleosome formation. However, the requirement of H3.3 nucleosomes and the functional crosstalk between KAP1 and H3.3 loading suggest that H3.3 might be necessary to suppress retroviral gene expression in mouse ESCs^96^. In line with this hypothesis, such interstitial heterochromatin regions in ESCs exhibit highly dynamic properties and rapid histone exchange, suggesting that H3.3 may control heterochromatin accessibility for efficient ERV repression^99,100^.

#### Plasticity and redundancy in H3.3 chaperone functions

Two H3.3 chaperone complexes perform unique functions in chromatin organization and gene regulation despite both modulating the H3.3 landscape. The unique functions are achieved by the tight control of the H3.3 supply via the preservation of H3.3/H4 in two spatially segregated compartments: the H3.3 pool in soluble (ASF1-HIRA pathway) and insoluble (PML-NB-DAXX pathway) contexts. However, accumulating reports show significant functional crosstalk between HIRA and DAXX (Fig. 3).

The newly synthesized H3.1/H4, H3.2/H4, and H3.3/H4 dimers are sequentially transferred through multiple protein chains to ASF1, which acts as a traffic hub for a stable supply of H3/H4 to the downstream chaperones CAF-1, HIRA, and DAXX. ASF1 has been shown to interact directly and indirectly with all three chaperones and contributes to functional crossover, likely due to its role in the maintenance and distribution of H3/H4 into distinct compartments^4,5^. Interestingly, HIRA, but not DAXX, can take the place of CAF-1 to maintain chromatin integrity during DNA replication. In instances where CAF-1 activity is compromised, HIRA can compensate by incorporating H3.3 into a broad range of genomes^22,101^. Furthermore, CenH3 overexpression leads to DAXX-dependent deposition of H3.3/H4-CenH3/H4 heterotetramers into euchromatin^102^, showing that DAXX has a certain level of flexibility in H3 binding and routing. Interestingly, ASF1 also assists DAXX/ATRX-mediated H3.3 deposition through PML-NBs. For example, ASF1 facilitates H3.3 enrichment in PML-NBs. The *Drosophila* DAXX-like protein (DLP) associated with ASF1 for H3.3 deposition in heterochromatin^103^. Consistently, proteomic analysis of the histone chaperone interaction network indicated that ASF1b acts partially upstream of DAXX for H3.3 deposition in heterochromatin^58^. Notably, the absence of DAXX improves the nucleosomal insertion of H3.3 through HIRA and CAF-1^53^, implying that two distinct compartments are interconnected via ASF1, which supervises the relative partitioning of H3.3/H4 as a distribution hub.

Furthermore, DAXX sequestration within PML-NBs is dynamically regulated under various circumstances, which can affect the H3.3 landscape. For example, upon heat shock or stress, the translocation of DAXX from PML-NB to centromeres and pericentromeres is increased to stimulate H3.3 deposition and RNA transcription^104^. In contrast, PML loss was shown to change the H3.3 deposition route from DAXX/ATRX-dependent to HIRA-dependent and the epigenetic features from H3K9me3 to H3K27me3 in PML-associated heterochromatin^56^. Moreover, DEK, a PML-associated protein, has been shown to control the balance of H3.3 loading between DAXX/ATRX and HIRA in somatic and ES cells. DEK depletion shifted the balance from DAXX/ATRX to HIRA, resulting in H3.3 occurring widely in the chromosome arms away from telomeres^105^. These studies suggest that PML-NBs and associated proteins play a role in the fine control of the H3.3 landscape by modulating interactions between H3.3 and its chaperoning partners. In addition, HIRA and ASF1 have been shown to accumulate in PML-NBs under certain conditions such as senescence^18,31^ and immune response to viral infection^106–109^. The unexpected concomitance of HIRA and DAXX in PML-NBs suggests their overlapping functions in chromatin regulation.

HIRA and DAXX function in chromatin function and gene regulation is somewhat redundant. Recent findings revealed that the HIRA complex is responsible for silencing specific genomic loci, such as MERV-L, by regulating H3.3 deposition and H3K9 methylation (Fig. 3b)^110^. In ESCs, histone chaperones may target and repress different classes of ERVs; HIRA regulates class III ERVs, while DAXX/ATRX regulates class I and II ERVs^95^. Furthermore, PML, DAXX, and HIRA are involved in the epigenetic repression of viral genes. Notably, HIRA relocalizes to PML-NBs after viral infection to promote heterochromatinization by H3.3 loading, viral gene silencing, and antiviral immunity^106–108^. During the inflammatory response, HIRA accumulates in PML-NB in a SUMO-dependent manner to mediate interferon-stimulated gene expression by H3.3 loading^109^, although it remains to be elucidated whether SUMOylation and PML-NB localization are necessary for genomic targeting. In addition, DAXX and HIRA exhibit a certain level of redundancy; for example, the DAXX/ATRX and HIRA complexes can partially compensate for each other’s absence in H3.3 deposition on the viral genome^106^. Similarly, HIRA can compensate for the loss of DAXX/ATRX by depositing H3.3 onto telomeric heterochromatin in alternative lengthening of telomeres (ALT) cancer cells^28^. Furthermore, DAXX/ATRX is implicated in gene activation. DAXX drives H3.3 deposition in regulatory regions of activity-responsive genes upon neuronal activation^111^. ATRX also upregulates the expression of ancestral pseudoautosomal region (aPAR) genes in the mouse forebrain by facilitating transcriptional elongation through G-rich sequences^112^. ATRX has a high affinity for G-quadraplexes, often formed in telomeres and gene regulatory regions upon DNA melting. Here, ATRX mediates H3.3 deposition to dissolve intragenic G-quadruplexes and facilitate the progression of polymerase II through G-rich regions.

### H3.3 chaperones in physiology and pathology

**Fig. 4:**
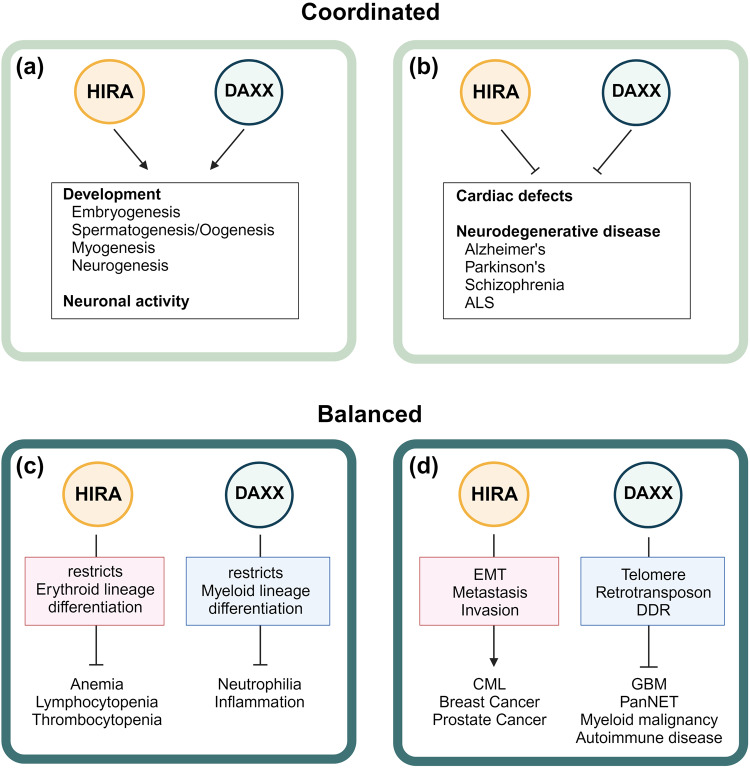
Coordinated or balanced activities of HIRA and DAXX in physiology and pathology. **a**, **b** HIRA and DAXX achieve a common goal despite targeting different genomic loci for H3.3 deposition. Such coordinately regulated H3.3 distribution by HIRA and DAXX maintains diverse cellular functions, including development and neuronal activity (**a**). In addition, HIRA and DAXX collaborate to prevent the development of diseases, including cardiac and neurodegenerative diseases (**b**). **c**, **d** In certain cellular contexts, the delicate balance between HIRA and DAXX is needed for normal physiology, and the disruption of the equilibrium can give rise to various pathological outcomes. For instance, HIRA and DAXX limit differentiation toward erythroid or myeloid lineages, respectively, maintaining the balance of hematopoiesis (**c**). Dysregulation of either HIRA or DAXX can result in hematological abnormalities. Furthermore, HIRA promotes oncogenesis by upregulating EMT, whereas DAXX acts as a tumor suppressor by protecting telomeres, retrotransposons, and DNA damage responses (DDR), demonstrating their competitive functions (**d**).

Due to their complex molecular mechanisms and myriad of functions, HIRA and DAXX have garnered considerable attention. Their roles in normal physiology, as well as numerous diseases, such as developmental disorders, cancer, and age-related diseases, have been extensively studied. In this section, we discuss the importance of HIRA and DAXX in disease, as well as their distinct and overlapping contributions (Fig. 4).

#### Developmental functions and disorders

H3.3 is essential in developmental processes, as demonstrated by the developmental retardation and early embryonic lethality of *H3f3a* and *H3f3b* double KO mice^113^. A lack of H3.3 results in reduced cell proliferation, increased cell death, and mitotic defects in a p53-dependent manner in mouse embryos^113^, and a low nucleosome turnover rate, improper lineage specification despite functional pluripotency, and upregulated expression of trophectoderm markers in mouse ESCs^114^. In line with this, mice lacking HIRA, DAXX, or ATRX are all embryonic lethal, demonstrating the importance of proper H3.3 distribution in development^115,116^. The absence of HIRA or DAXX leads to defects in oogenesis, spermatogenesis, and embryogenesis^117^ (Fig. 4a). Loss of ATRX results in defects in trophoblast development and the pattern of the X-inactivation center in extraembryonic tissue^118^. DAXX regulates the expression of PRC1 target genes^119^, while HIRA deposits H3.3 at bivalent promoters^14^, enriched with PRC2 and H3K27me3. Knockdown of H3.3 or HIRA results in decreased H3K27me3 levels and increased H3K27Ac levels, leading to activation of bivalent genes and ESC differentiation^14,114^.

Furthermore, the HIRA and DAXX complex is involved in multiple lineage specifications, including myogenesis, neurogenesis, and hematopoiesis. In muscle stem cells, HIRA and DAXX unidirectionally maintain muscle lineage identity (Fig. 4a). Cabin1 within the HIRA complex recruits Suv39h1, and HDACs, and inhibits myogenic genes and alternative cell fate-determining genes^29,30^. DAXX simultaneously inhibits E2A-dependent expression of key myogenic genes^120^. Upon differentiation que, HIRA and DAXX drive myogenesis. PML-NB are reduced, and DAXX is translocated to chromocenters to maintain the structural organization of the heterochromatin domain^92,93^. HIRA complexes undergo rearrangement to form an activator complex with p300 and/or Asf1 for terminal differentiation^71^. Such importance of HIRA and DAXX in muscle development provides a rationale for cardiac muscle defects associated with HIRA and DAXX (Fig. 4b). Importance of HIRA in heart physiology is evidenced by impaired heart development in HIRA KO mice, dependence of mESC cardiac differentiation on HIRA-mediated H3.3 deposition^121,122^, and abnormal low expression of the HIRA gene in the myocardium of patients with tetralogy of Fallot^123^. In addition, DAXX is associated with myocardial ischemia/reperfusion-mediated proapoptotic signaling in mice^124^.

During neurogenesis, both HIRA and ATRX play an essential role in maintaining a pool of neural progenitor cells (NPCs) and contribute to embryonic brain development. The involvement of HIRA in NPC self-renewal is mediated through its WD40 domain^125^, which is shared by the proteins crucial for brain development and neuronal connectivity^126^. Furthermore, ATRX depletion leads to increased cell cycle exit and premature differentiation of NPCs^127^, while PML is expressed in NPCs but not in differentiated neurons^128^. These results suggest the importance of H3.3 loading by chaperones of the PML/DAXX/ATRX complex in neurogenesis^129^. The importance of proper distribution of H3.3 is supported by the pathological consequences arising from deregulated H3.3 and its chaperone activities. For example, patients with de novo truncating variants of HIRA may experience neurodevelopmental disorders^130^. Furthermore, PML loss results in altered corticogenesis and smaller brains^128^. The de novo missense variants of *H3F3A* and *H3F3B*, which disrupt interactions with DNA, other histones, and histone chaperone proteins, are associated with neurodevelopmental delay and neurologic abnormalities^131^.

Although the functional consequences of DAXX and HIRA were concomitant in myogenesis and neurogenesis (Fig. 4a), their effects are distinct in hematopoiesis. Loss of H3.3 in hematopoietic stem cells (HSCs) downregulates the expression of stemness- and lineage-specific genes with a predominant increase in H3K27me3 levels. In contrast, H3K9me3 level are reduced in ERV repeats, and chromatin accessibility is increased in the interferon regulatory regions. Therefore, a delicate interplay between H3K27me3 and H3K9me3 marks maintains the adaptability of HSC chromatin^132^. Similarly, the balance between HIRA and DAXX complexes appears to be critical in the determination of the hematopoietic lineage (Fig. 4c). DAXX represses myeloid lineages^133^, while HIRA restricts erythroid-biased differentiation^134^. Mice with hematopoietic-specific deletion of HIRA show thrombocytopenia, anemia, and lymphocytopenia^135^. DAXX-KO mice develop neutrophilia and inflammation^133^. These pathological consequences highlight the critical role of the balanced regulation of H3.3 by HIRA and DAXX complexes in normal hematopoiesis and immune system development.

#### Cancer

Recurrent and distinct mutations in H3.3 have been implicated in cancers. These mutations are often found in the N-terminal tail of H3.3, a region enriched with PTMs. In particular, mutations of K27, G34, and K36 of H3.3 are frequently observed in various cancers. The H3.3K27M mutant is found in 78% of diffuse intrinsic pontine gliomas and, in some cases, high-grade astrocytomas^136,137^. The mutation in H3.3G34 affects H3.3K36 modification^138,139^. Interestingly, distinct substituted amino acid residues are found in specific cancers: G34V in glioma, G34R in pediatric high-grade glioma, G34W or G34L in 92% of giant cell tumors of bone^140^, and H3.3K36 mutation in 95% of chondroblastoma and sometimes in clear-cell chondrosarcoma. These mutations exert a profound influence on global epigenetic states despite the presence of H3.1, highlighting the critical role of H3.3 in the epigenetic landscape in cancer.

Accordingly, dysregulation of HIRA or DAXX complexes is observed in various types of cancers, such as breast, lung, prostate, colorectal, glioma, pancreatic, leukemia, prostate, gastric, ovarian, and chondrosarcoma. In contrast to development processes, altered functions of HIRA and DAXX as H3.3 histone chaperones have distinct consequences in tumor development and progression (Fig. 4d). HIRA plays oncogenic roles by depositing H3.3 in promoters related to epithelial-mesenchymal transition, metastasis and invasion in chronic myeloid leukemia, breast cancer, and prostate cancer^101,141^. In contrast, the incorporation of H3.3 by DAXX suppresses cancer development by maintaining telomeres and promoting the DNA damage response. Dysfunctional DAXX and ATRX result in homologous recombination of telomeres, which leads to ALT^142^. Consistently, DAXX and ATRX loss-of-function mutations have been reported in various ALT-positive cancers, such as pancreatic neuroendocrine tumors (PanNETs) and pediatric glioblastoma multiforme (GBM)^143–145^. In addition to telomere maintenance, loss-of-function mutations in DAXX and ATRX lead to defective silencing of retrotransposable elements, leading to chronic inflammation, myeloid malignancies, and autoimmune diseases^146–148^. Recurrent DAXX mutations are observed in thyroid carcinoma^149^, while DAXX degradation by SPOP contributes to kidney tumorigenesis^150^.

H3.3 chaperone-independent activities of HIRA and DAXX lead to quite different consequences. In contrast to the oncogenic consequences of HIRA-mediated H3.3 deposition, HIRA can function as a tumor suppressor independent of its chaperone activity. HIRA, together with ASF1, can aid in macroH2A deposition and is needed for SAHFs and cell cycle exit^18,31^. Involvement of HIRA in cell cycle regulation can be further inferred by cyclin/CDK2-mediated phosphorylation, and S-phase arrest upon ectopic expression of HIRA^151^. The activated senescence phenotype serves as a tumor-suppressive mechanism in a mouse skin cancer model^152^. Furthermore, the loss of HIRA activates MYC and its target genes, independent of its chaperone activity, and enhances the proliferation and invasion of fumarate hydratase deficient hereditary leiomyomatosis and renal cell carcinoma (HLRCC)^153^.

The chaperone-independent activity of DAXX exerts oncogenic effects, as opposed to the tumor-suppressive effects of its chaperone activity. DAXX interacts with and modulates many transcription factors. DAXX interacts with CCAAT/enhancer-binding protein beta (CEBP-b) and activates the ERK signaling pathway to induce the formation of ovarian cancer ascites^154^. Furthermore, DAXX functions as an anaphase-promoting complex (APC) inhibitor, promoting chromosome instability and cancer predisposition during prostate cancer development^155^. Highly expressed DAXX can promote cell proliferation and chemoresistance in ovarian cancer^156^. In addition, in prostate cancer, downregulation of the autophagy modulators DAPK3 and ULK1 by DAXX enhances tumor growth and primary prostatic malignancy^157^. In cancers with CENP-A overexpression, DAXX aberrantly deposits CENP-A into noncentromeric DNA, including the 8q24 locus, which can overexpress the MYC gene^158^.

#### Neurodegenerative disorders

In addition to their important roles in neuronal development, HIRA and DAXX actively participate in adult neuronal activities. Unlike other postreplicative cells, H3.3 remains highly dynamic in adult neurons, and the HIRA-dependent turnover of H3.3 is essential in neuronal plasticity^159^. Furthermore, DAXX promotes H3.3 loading to activate immediate early genes in the cortical neurons of the mouse CNS^111^. Consequently, dysregulation of HIRA has been implicated in the alteration of chromatin remodeling and gene expression patterns in Alzheimer’s disease. Likewise, abnormal DAXX activity contributes to protein aggregation and neuronal dysfunction in Parkinson’s disease^160^, schizophrenia^161^, and amyotrophic lateral sclerosis. Independent of its function as a histone chaperone, DAXX has been reported to prevent and reverse the aggregation of neurodegeneration-associated proteins^162^. In addition, upregulation of DAXX by the proinflammatory cytokine IFN-gamma upon tissue damage or infection regulates the population of activated microglia by mediating the apoptosis of microglia^163,164^. However, loss of DAXX fails to control activated microglia, contributing to the pathogenesis of neurodegenerative disorders^165^. Altogether, both HIRA and DAXX maintain healthy neuronal activity, and loss of function of either protein can promote the development of neurodegenerative disorders.

#### Senescence

Despite the limited proliferation capacity in senescent cells, the landscape of chromatin is maintained by depositing newly synthesized histones dynamically^166^. Histone H3.3 displaces H3.1 and H3.2 during senescence, leading to the relaxation of chromatin and transcription activation at specific loci. The active promoters in senescent cells are enriched in H4K16ac, which requires HIRA for incorporation^152^. In addition to HIRA, ATRX is also needed for SAHF formation. PML induced by oncogenic Ras promotes premature senescence^167^. In contrast, DAXX mRNA expression levels decrease with aging in various tissues, such as the kidney, heart, and cortex^168^. The impaired function of DAXX/ATRX in telomere maintenance results in telomere shortening and cell senescence. Furthermore, independent of its chaperone activity, DAXX suppresses p53/p21-mediated senescence in mouse ovarian epithelial cells^169,170^. Thus, it is likely that DAXX plays multifaceted roles in senescence, depending on its interacting proteins and cellular contexts.

## Conclusions and prospects

As we explore the diverse functions of H3.3 chaperone complexes in this review, intriguing questions arise regarding the mechanisms underlying their similar but distinct actions. One intriguing question is what dictates the deposition route of H3.3 into specific chromatin domains. Determining whether the availability of H3.3 in various storage compartments could predispose its utilization in different genomic regions will help answer this question. In addition, further research is required to fully elucidate the function of H3.3-containing nucleosomes in chromatin contexts, especially where domain features are primarily attributed to histone marks. It is also of great interest to determine what intrinsic properties of H3.3-containing nucleosome enable it to function in both euchromatin and heterochromatin, thereby contributing to its adaptability in a variety of chromatin environments. Moreover, investigating the genomic and epigenomic contexts that determine the dependence of DAXX on ATRX and vice versa would shed light on the intricate interplay between the chaperone-dependent and chaperone-independent functions of these proteins in chromatin regulation.

We have highlighted the importance of the delicate equilibrium between H3.3 histone chaperones for maintaining normal physiological and cellular functions. Any disturbances to this equilibrium can result in various pathological outcomes. To fully comprehend the intricate interplay between histone chaperone complexes, additional research is needed. The development of small molecules that modulate the activities of HIRA or DAXX complexes could provide valuable insights and lead to therapeutic applications for cancer and other diseases. By addressing these questions and investigating the complex dynamics of the H3.3 landscape, new avenues will be revealed for understanding chromatin regulation and its role in health and disease.
